# Non‐Invasive Assessment of Perineural Invasion Density: A Novel Marker for Prognosis and Treatment Stratification in Prostate Cancer

**DOI:** 10.1002/advs.78460

**Published:** 2026-10-31

**Authors:** Bohao Liu, Long Zhang, Weian Zhu, Meng Yang, Xiang Liu, Xiaoyang Li, Peng Qin, Zhengxu Lin, Qian Cai, Jiayu Zheng, Xiao Zhao, Chen Zou, Xuan Wen, Longhao Xu, Daqin Wu, Lin Zhang, Cuilian Zhang, Kaiwen Li, Chunhui Wang, Wenlong Zhong, Yun Luo

**Affiliations:** ^1^ Department of Urology The Third Affiliated Hospital of Sun Yat‐sen University Guangzhou China; ^2^ Department of Pathology Sun Yat‐sen Memorial Hospital Sun Yat‐sen University Guangzhou China; ^3^ Department of Urology Yan'an Hospital Affiliated to Kunming Medical University Kunming Medical University Kunming China; ^4^ Department of Radiology Sun Yat‐sen Memorial Hospital Sun Yat‐sen University Guangzhou China; ^5^ Department of Urology Peking University People's Hospital Peking University Beijing China; ^6^ Department of Urology Sun Yat‐sen Memorial Hospital Sun Yat‐sen University Guangzhou China

**Keywords:** artificial intelligence, perineural invasion, prognosis, prostate cancer

## Abstract

We established and validated perineural invasion density (PNID) as a superior prognostic marker and developed an MRI–based AI model for its preoperative prediction. In this multicenter study of 1091 PCa patients undergoing radical prostatectomy, we evaluated PNID's association with biochemical recurrence (BCR) and radiographic progression–free survival (rPFS). We also analyzed concurrent β–blocker or antimuscarinic use with adjuvant ADT. A deep learning model was developed in a primary cohort (n = 523) and validated in two external cohorts (n = 317, 251). High–PNID was an independent prognostic factor for BCR (HR 4.06, 95% CI: 2.75–5.97, P < 0.001) and rPFS (HR 4.61, 95% CI: 2.97–7.16, P < 0.001). Notably, concurrent β–blocker use was significantly associated with favorable BCR outcomes exclusively in the High–PNID subgroup (HR 0.32, 95% CI: 0.12–0.84, P = 0.021), with no benefit in the Non–High PNID population or with antimuscarinics. The AI model showed excellent performance predicting High–PNID, with AUCs of 0.917 (training), 0.842 (internal validation), 0.889 and 0.915 (external validations). Model–identified High–PNID patients had significantly worse outcomes. PNID surpasses traditional PNI in prognostic accuracy and identifies a high–risk phenotype. High–PNID may define a subgroup benefiting from adjuvant β–blocker therapy, providing an exploratory clinical rationale for targeting the nerve–tumor microenvironment in stratified subpopulations. Hence, this model may be a reliable, non–invasive tool for preoperative identification of this high–risk subgroup, aiding clinicians in personalized risk stratification and treatment planning.

## Graphical abstract

This study establishes perineural invasion density (PNID) as a superior prognostic marker in prostate cancer, outperforming conventional perineural invasion in predicting biochemical recurrence and radiographic progression. Concurrent β–blocker use is associated with favorable outcomes exclusively in High–PNID patients. An MRI–based deep learning model non–invasively identifies this high–risk phenotype preoperatively, supporting personalized risk stratification and providing an exploratory rationale for targeting the nerve–tumor microenvironment.

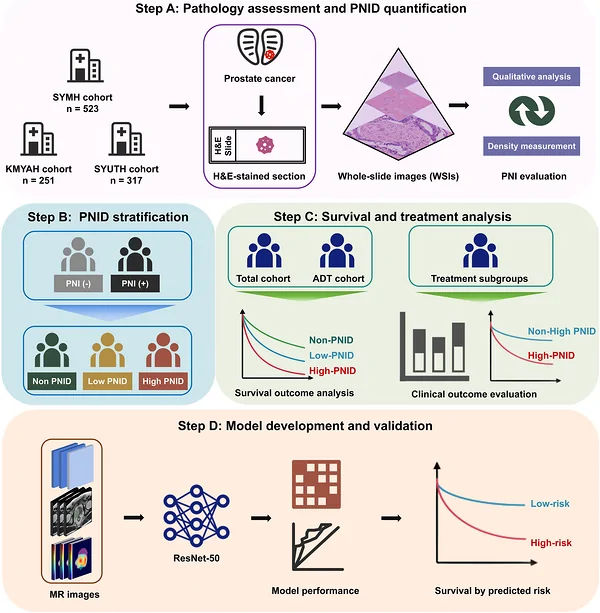

## 1 Introduction

Prostate cancer (PCa) represents the second most common malignancy in men worldwide [1]. The prostate gland is richly innervated by nerve fibers, which have been increasingly recognized as essential components of the tumor microenvironment that facilitate cancer progression and dissemination [2, 3]. Perineural invasion (PNI) represents the most direct and clinically observable manifestation of cancer–nerve interplay, occurring in 17%–84% of PCa patients in surgical or biopsy specimens [4, 5]. Although considered a distinct route for tumor dissemination and an adverse pathological feature, the prognostic value of PNI in PCa remains controversial. While some studies reported that PNI independently predicted biochemical recurrence (BCR) and progression [6, 7, 8], others found no significant association after adjusting for Gleason score and pathological stage [9, 10]. This controversy likely stems from the limitation of binary classification, which fails to capture the extent of neural involvement and may obscure the true biological impact of PNI.

Quantitative assessment of PNI offers a promising avenue for more precise risk stratification by reflecting the degree rather than mere presence of neural invasion [11, 12]. However, clinical implementation is currently hindered by methodological inconsistencies regarding evaluation standards and optimal cutoff values [12, 13]. Furthermore, accurate quantification predominantly relies on post–operative surgical specimens, as biopsy often underestimates PNI burden due to limited sampling [11, 14]. This critical prognostic information can thus only be reliably determined post–operatively. Therefore, establishing a standardized, reproducible quantitative metric of PNI and exploring non–invasive predictive strategies are urgently warranted.

Artificial intelligence (AI) has emerged as a powerful tool for PCa precision medicine, autonomously extracting high–dimensional imaging features to support clinical decision–making [15]. Magnetic resonance imaging (MRI) based deep learning models have demonstrated remarkable efficacy in non–invasively predicting complex biological heterogeneity, including molecular subtypes, prognosis, and treatment responses across various malignancies [16, 17, 18, 19]. Notably, these approaches show great promise in non–invasively characterizing critical pathological features [20, 21]. However, the integration of these advanced modalities specifically for the refined assessment of PNI in PCa warrants further investigation.

Herein, we conducted a large–scale multicenter study to address these gaps. First, we propose and validate perineural invasion density (PNID) as a novel, standardized quantitative metric designed to more accurately reflect the intensity of tumor–nerve interaction. Second, we investigate whether high PNID status can identify a biological subset of patients who may derive specific benefit from adjuvant therapy targeting the nerve–tumor microenvironment, such as concurrent β–blockers. Finally, to bridge the gap to clinical utility, we develop and validate an AI–driven model based on preoperative MRI to non–invasively predict High–PNID status, enabling risk stratification and potential treatment tailoring before surgery.

## 2 Methods

### 2.1 Patient Cohorts

We conducted this multicenter cohort study at Sun Yat–sen Memorial Hospital (SYMH), the Third Affiliated Hospital of Sun Yat–sen University (SYUTH), and Kunming Medical University Affiliated Yan'an Hospital (KMYAH) as part of a registered clinical trial (ChiCTR2500101475). The inclusion criteria were: (1) histologically confirmed PCa; (2) treatment with radical prostatectomy (RP); and (3) availability of complete clinicopathological and follow–up data. The exclusion criteria were: (1) lack of evaluable preoperative pelvic MRI; (2) unclear hematoxylin and eosin (H&E) pathological slides; (3) malignancies not originating from the prostate. An overview of the study design is shown in Figure 1. This study was approved by the Institutional Review Boards of SYMH (SYSKY–2025–678–01), SYUTH (II2025–096–01), and KMYAH (2025–132–01). Informed consent was waived due to the retrospective design. All procedures were conducted in accordance with the ethical principles outlined in the Declaration of Helsinki.

**FIGURE 1:**
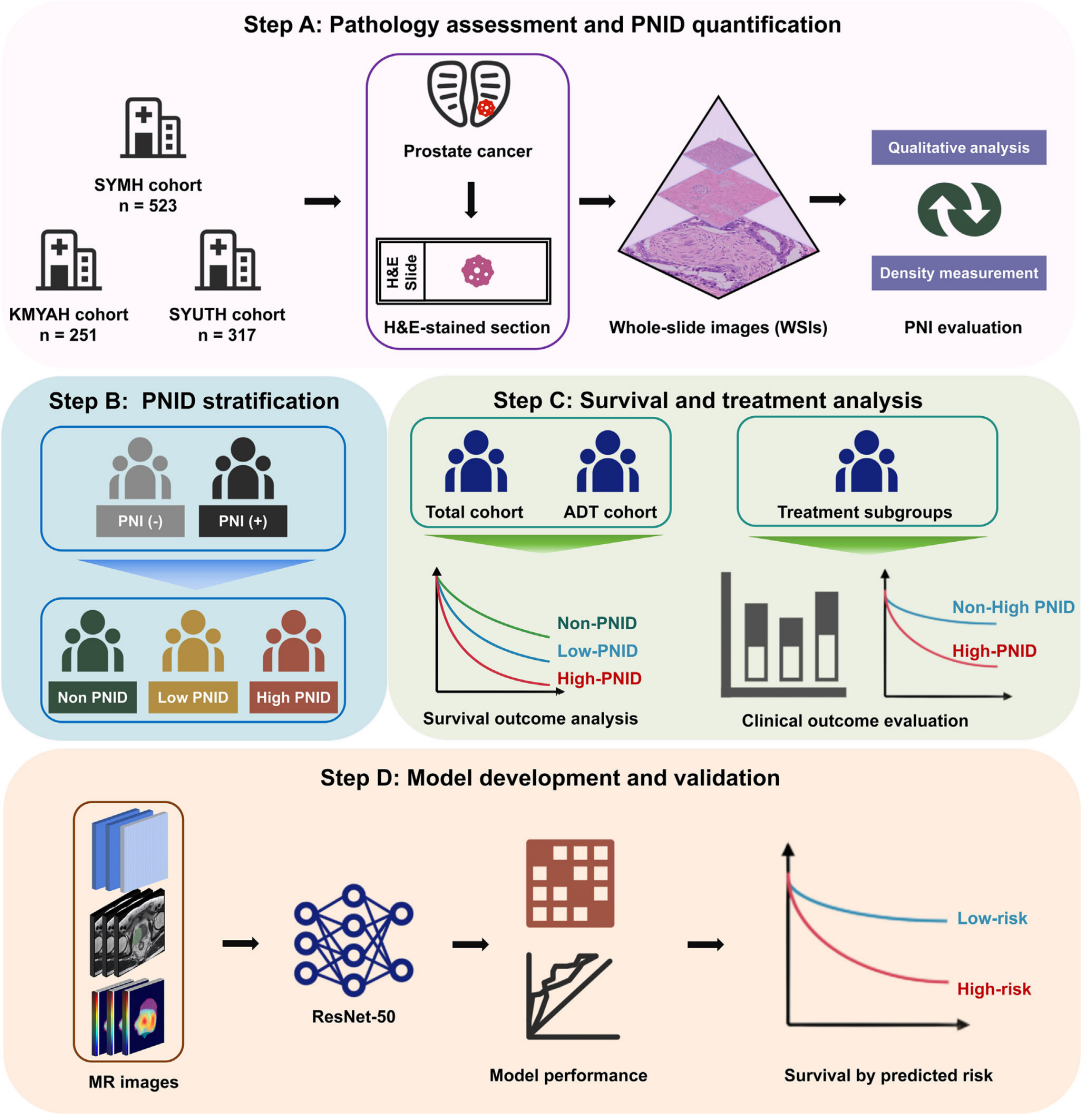
Overview of the study design. PNI, perineural invasion; PNID, perineural invasion density; WSI, whole–slide image; MR, magnetic resonance.

We retrospectively collected clinicopathological variables, including age, body mass index (BMI), total prostate –specific antigen (tPSA), Prostate Imaging–Reporting and Data System (PI–RADS) score, International Society of Urological Pathology (ISUP) group, pathological tumor and nodal stage (pT and pN), and the presence of PNI, positive surgical margins (PSM), and lymphovascular invasion (LVI). BCR was defined as a serum PSA level of ≥ 0.2 ng/mL on two consecutive measurements following RP [22]. Radiographic progression–free survival (rPFS) was defined as the time from RP to radiographic disease progression (adapted from PCWG3 criteria for progression assessment [23]) or death from any cause, whichever occurred first.

### 2.2 Definition, Evaluation, and Prognostic Value of PNID

PNI was defined as the presence of tumor cells in close proximity to a nerve with involvement of at least one–third of its circumference, or tumor infiltration into any layer of the nerve sheath [24]. To quantify the extent of PNI and better assess its clinical significance, we introduced a novel parameter termed PNID. The method for assessing PNID was as follows: the H&E–stained section with the deepest tumor invasion was selected, and a whole slide image (WSI) was generated by scanning at 20× magnification. The invasive front was examined on the WSI to identify the area with the highest density of PNI (hotspot). Within a 1 mm^2^ square area selected in the hotspot, the number of nerves with and without PNI were both counted. PNID was calculated by dividing the number of nerves with PNI by the total number of nerves within this square area and multiplying by 100%. The formula for PNID is as follows:

$$\mathrm{PNID}=\frac{\mathrm{Number}\;\mathrm{of}\;\mathrm{nerves}\;\mathrm{with}\;\mathrm{PNI}}{\mathrm{Total}\;\mathrm{number}\;\mathrm{of}\;\mathrm{nerves}}\;\times \;100\%$$

Dual–independent PNID assessments were performed by two pathologists, who were blinded to all clinical and survival outcomes. The intraclass correlation coefficient (ICC) for inter–observer reproducibility was 0.977 (*P* < 0.001), indicating excellent agreement. To corroborate the morphological fidelity of H&E–based PNID assessment, multiplex immunofluorescence staining was conducted on representative specimens (Method S1).

Among PNI–positive cases, the median PNID value was determined and used as the cutoff threshold to dichotomize patients into two subgroups: hHigh–PNID (≥ 50%) and Low–PNID (< 50%). Consequently, all patients were stratified into three groups based on PNID status for subsequent survival analyses: Non–PNID (PNI–negative), low PNID, and high PNID. Kaplan–Meier survival curves were generated separately for each of the three cohorts. Cox proportional hazards regression was then performed in the overall cohort to evaluate the prognostic significance of PNI and PNID. Sankey diagrams were used to visualize the flow and distributions of patients stratified by PNI status, PNID status, and survival outcomes.

To establish the incremental prognostic value of PNID over existing pathological factors, nested Cox proportional hazards models were evaluated with Harrell's C–index, likelihood ratio tests, and Akaike Information Criterion (AIC) (Method S2).

### 2.3 Treatment Subgroup Analysis in the ADT Cohort

To evaluate the potential therapeutic impact of neuro–signaling inhibitors, a subgroup analysis was performed on the initial cohort of 1091 patients. Inclusion criteria required the initiation of combined androgen deprivation therapy (ADT), defined as the concurrent use of luteinizing hormone–releasing hormone (LHRH) agonists and androgen receptor (AR) inhibitors, within 2 weeks after RP. To strictly evaluate the efficacy of combination therapy and avoid immortal time bias, patients with a follow–up duration of less than 6 months or those who experienced BCR within the first 6 months were excluded. Based on these criteria, a total of 309 patients were included in the final ADT cohort.

Drug exposure was defined using WHO ATC classification codes. β–blocker exposure was defined as the use of systemic agents (selective or non–selective) for at least 6 months, excluding ophthalmic formulations [25]. Antimuscarinic exposure was defined as the postoperative use of agents indicated for overactive bladder for at least 3 months [26]. Detailed pharmacological classifications and ATC codes are provided in Table S1. Patients receiving ADT without meeting these medication exposure thresholds were categorized as the control group (ADT alone). Given the limited sample size, patients receiving concurrent β–blockers and antimuscarinics were included in the treatment arm of both respective analyses.

Prior to therapeutic evaluation, we validated the prognostic value of PNID within the ADT cohort (n = 309) to determine the optimal risk stratification strategy. Kaplan–Meier survival analysis was first performed using the trichotomous PNID status. Based on the prognostic consistency where Low–PNID outcomes closely paralleled those of Non–PNID, patients were subsequently dichotomized into High–PNID and Non–High PNID for the therapeutic interaction analysis.

Comparative analyses were then conducted to evaluate the BCR–free survival between treatment arms: (1) ADT alone versus ADT plus β–blockers; and (2) ADT alone versus ADT plus antimuscarinics. Subgroup analyses were stratified into High–PNID and Non–High PNID groups to explore potential effect modification. Survival outcomes were visualized using Kaplan–Meier curves with log–rank tests, and hazard ratios (HRs) were estimated using Cox proportional hazards models to quantify the effect size of the combined therapy in each stratum.

### 2.4 Development and Validation of an AI–Based Model for PNID Prediction

Non–fat–saturated axial T2–weighted MR images were acquired, converted to Nearly Raw Raster Data (NRRD) format, and preprocessed via bilinear interpolation to a resolution of 256 × 256 pixels. Regions of interest (ROIs) were manually delineated using 3D Slicer software (version 5.2.1) by two urological radiologists (5 and 15 years of experience) who were blinded to clinical data. For each case, the axial slice demonstrating the maximal tumor cross–sectional area was selected as the model input. In accordance with CLAIM guidelines to prevent data leakage [27], cohort partitioning was established prior to model development, with preprocessing performed independently within each set and external validation cohorts remaining strictly isolated throughout.

The PNID prediction model was developed utilizing the ResNet–50 framework initialized with ImageNet pre–trained parameters. Architectural modifications included replacing the terminal fully connected layer with a linear transformation layer featuring a single output neuron. We employed BCEWithLogitsLoss as the loss function and the Adam optimizer with L2 regularization (weight decay = 1 × 10^−6^). The model was implemented using PyTorch (version 2.8.0 + cu129) on an NVIDIA GeForce RTX 4060 GPU. Training parameters included a batch size of 64, an initial learning rate of 0.0005 (decayed by 0.1 every 50 epochs), and a maximum of 200 epochs with early stopping triggered after 50 epochs of no improvement.

Model performance was comprehensively evaluated using metrics such as overall accuracy, area under the receiver operating characteristic curve (AUC), sensitivity, specificity, positive predictive value (PPV), and negative predictive value (NPV), visualized through ROC curves and confusion matrices. A guided gradient–weighted class activation mapping (Guided Grad–CAM) approach was employed to interpret the model by visualizing key regions within the ROI contributing to predictions. Additionally, Kaplan–Meier survival analysis was performed based on model–predicted PNID status to assess prognostic stratification. All analyses were performed in Python (version 3.12.4, Anaconda distribution) using Jupyter Notebook. The complete code is available at https://github.com/BohaoLiu1998/resnet50_for_PNID.

### 2.5 Statistical Analysis

Continuous variables were presented as mean ± standard deviation or median (interquartile range [IQR]), and compared using one–way ANOVA, Kruskal–Wallis test, or Mann–Whitney U test, as appropriate. Categorical variables were compared using the Chi–square test or Fisher's exact test. Survival outcomes were estimated via the Kaplan–Meier curves, log–rank tests, and Cox proportional–hazards model. To identify independent prognostic factors, both univariable and multivariable Cox proportional hazards regression models were employed. Model discrimination and performance were further evaluated using ROC curve analysis and confusion matrices. All statistical analyses were performed using R software (version 4.3.1). A two–sided *P* < 0.05 was considered statistically significant.

## 3 Results

### 3.1 Patient Characteristics

A total of 1665 patients were initially screened, of whom 1091 were ultimately enrolled in the study. The detailed flowchart of patient selection is presented in Figure S1. The final cohort included patients diagnosed with PCa between July 2014 and August 2025 from SYMH (n = 523), SYUTH (n = 317), and KMYAH (n = 251). In the overall cohort, PNI was identified in 611 (56.0%) patients, while 480 (44.0%) were PNI–negative. Further stratification based on PNID classified 302 patients (27.7%) as Low–PNID and 309 (28.3%) as High–PNID. The baseline characteristics of the three cohorts are summarized in Table 1.

**TABLE 1:** Clinicopathological characteristics of patients in the total cohort and in individual cohorts.

Characteristics	Total	SYMH cohort	SYUTH cohort	KMYAH cohort
**N**	1091	523	317	251
**Age, median (IQR), years**	70 (64, 75)	68 (62, 72)	70 (64, 75)	75 (69, 81)
**BMI, median (IQR), kg/m^2^**	23.66 (21.67, 25.51)	23.66 (21.84, 25.54)	23.88 (21.48, 25.91)	23.44 (21.48, 25.07)
**tPSA, n (%)**				
< 10 ng/mL	367 (33.6%)	189 (36.1%)	91 (28.7%)	87 (34.7%)
10 – 20 ng/mL	324 (29.7%)	181 (34.6%)	86 (27.1%)	57 (22.7%)
> 20 ng/mL	400 (36.7%)	153 (29.3%)	140 (44.2%)	107 (42.6%)
**PI‐RADS score, n (%)**				
≤ 3	313 (28.7%)	113 (21.6%)	99 (31.2%)	101 (40.2%)
> 3	778 (71.3%)	410 (78.4%)	218 (68.8%)	150 (59.8%)
**pT, n (%)**				
12	646 (59.2%)	287 (54.9%)	189 (59.6%)	170 (67.7%)
3‐4	445 (40.8%)	236 (45.1%)	128 (40.4%)	81 (32.3%)
**pN, n (%)**				
0	940 (86.2%)	449 (85.9%)	258 (81.4%)	233 (92.8%)
1	151 (13.8%)	74 (14.1%)	59 (18.6%)	18 (7.2%)
**ISUP group, n (%)**				
1	161 (14.8%)	65 (12.4%)	50 (15.8%)	46 (18.3%)
2	299 (27.4%)	174 (33.3%)	53 (16.7%)	72 (28.7%)
3	225 (20.6%)	123 (23.5%)	47 (14.8%)	55 (21.9%)
4	199 (18.2%)	80 (15.3%)	76 (24.0%)	43 (17.1%)
5	207 (19%)	81 (15.5%)	91 (28.7%)	35 (13.9%)
**PSM, n (%)**				
Negative	640 (58.7%)	279 (53.3%)	193 (60.9%)	168 (66.9%)
Positive	451 (41.3%)	244 (46.7%)	124 (39.1%)	83 (33.1%)
**PNI, n (%)**				
Negative	480 (44%)	164 (31.4%)	147 (46.4%)	169 (67.3%)
Positive	611 (56%)	359 (68.6%)	170 (53.6%)	82 (32.7%)
**PNID, n (%)**				
Non	480 (44.0%)	164 (31.4%)	147 (46.4%)	169 (67.3%)
Low	302 (27.7%)	189 (36.1%)	76 (24.0%)	37 (14.7%)
High	309 (28.3%)	170 (32.5%)	94 (29.7%)	45 (17.9%)
**LVI, n (%)**				
Negative	953 (87.4%)	442 (84.5%)	267 (84.2%)	244 (97.2%)
Positive	138 (12.6%)	81 (15.5%)	50 (15.8%)	7 (2.8%)

BMI, body mass index; IQR, interquartile range; tPSA, total prostate‐specific antigen; PI‐RADS, Prostate Imaging Reporting and Data System; pT, pathological tumor stage; pN, pathological node stage; ISUP, International Society of Urological Pathology; PSM, positive surgical margin; PNI, perineural invasion; PNID, perineural invasion density; LVI, lymphovascular invasion; SYMH, Sun Yat‐sen Memorial Hospital; SYUTH, Third Affiliated Hospital of Sun Yat‐sen University; KMYAH, Kunming Medical University Affiliated Yan’ an Hospital.

### 3.2 PNID Demonstrates Superior Prognostic Value Compared to Qualitative PNI

We initially evaluated the prognostic efficacy of traditional qualitative PNI status, which revealed inconsistent risk stratification across endpoints. While qualitative PNI successfully predicted BCR across all cohorts (*P* < 0.05; Figure S2A–C), it failed to significantly distinguish rPFS outcomes in the SYMH cohort (*P* = 0.277; Figure S2D), despite showing significance in SYUTH and KMYAH cohorts (*P* < 0.001; Figure S2E,F). This instability was further confirmed by multivariate analysis in the total cohort: although PNI remained an independent risk factor for BCR (HR: 2.307; 95% CI: 1.598–3.330; *P* < 0.001; Table S2), it failed to demonstrate independent prognostic value for rPFS after adjusting for confounding factors (HR: 1.177; 95% CI: 0.864–1.603; *P* = 0.302; Table S3) in the total cohort. These inconsistencies suggest that the Qualitative PNI may be insufficient for precise risk stratification, particularly for long–term survival.

We re–classified the total cohort into three subgroups based on the H&E quantification of PNID status in PNI–positive patients (Figure 2A). Sankey diagrams visualized the patient flow across PNI status, clinical outcomes, and PNID status, showing that a considerable portion of PNI–positive patients remained free from BCR and radiographic progression (Figure 2B,C). Kaplan–Meier analyses consistently identified High–PNID as the poorest prognostic group. For BCR, High–PNID patients exhibited significantly lower survival rates compared to Non–PNID and Low–PNID groups in SYMH cohort and SYUTH cohort (all pairwise *P* < 0.001; Figure 2D,E), and KMYAH cohort (vs. Non: *P* < 0.001; vs. Low: *P* = 0.029; Figure 2F). Similarly, for rPFS, High–PNID status predicted rapid progression in SYMH cohort (vs. Non: *P* = 0.002; vs. Low: *P* < 0.001; Figure 2G), SYUTH and KMYAH cohorts (all pairwise *P* < 0.001; Figure 2H,I). Crucially, no significant survival difference was observed between Low–PNID and Non–PNID groups across any endpoint (*P* > 0.05), suggesting that Low–PNID carries a relatively benign prognosis comparable to Non–PNID. Furthermore, multiplex immunofluorescence staining of Pan–CK, S100, and DAPI illustrated the morphological characteristics of the three PNID subgroups (Figure S6).

**FIGURE 2:**
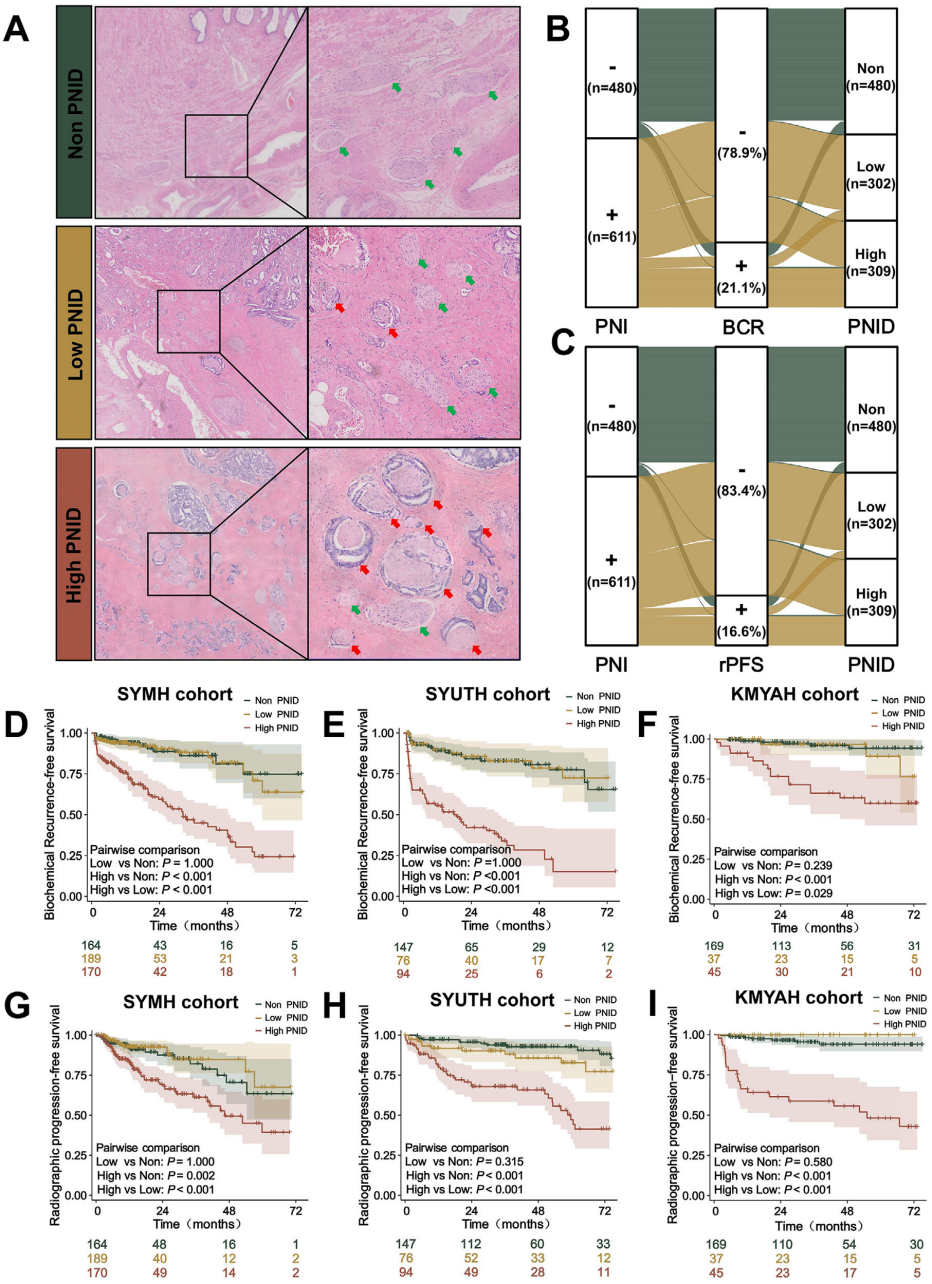
Correlation between PNID status and prognosis. (A) Representative H&E‐stained slides showing Non PNID, Low PNID and High PNID (20×). Red arrows indicate PNI sites; green arrows indicate nerves without invasion. (B) Sankey diagram illustrating the distributions of patients across PNI status, BCR status, and PNID status in the total cohort. (C) Sankey diagram illustrating the distributions of patients across PNI status, rPFS status, and PNID status in the total cohort. (D–I) Kaplan–Meier analysis of BCR and rPFS of patients with PCa stratified by the PNID status in the SYMH, SYUTH and KMYAH cohorts. *P* values were calculated using pairwise log‐rank tests. PNID, perineural invasion density; PNI, perineural invasion; BCR, Biochemical recurrence; rPFS, Radiographic progression‐free survival; SYMH, Sun Yat‐sen Memorial Hospital; SYUTH, Third Affiliated Hospital of Sun Yat‐sen University; KMYAH, Kunming Medical University Affiliated Yan’ an Hospital.

To comprehensively evaluate the clinical relevance of PNID status, we analyzed its association with clinicopathological features and subsequently validated its independent prognostic value using multivariate Cox regression. As shown in Table 2, higher PNID status correlated with increased tPSA levels, higher PI–RADS scores, more advanced pT and pN stages, higher ISUP grades, as well as higher frequencies of PSM and LVI across all three cohorts (all *P* < 0.05). Multivariate Cox regression confirmed High–PNID as an independent risk factor for both BCR (HR: 4.056; 95% CI: 2.754–5.973; *P* < 0.001; Table 3) and rPFS (HR: 4.609; 95% CI: 2.967–7.161; *P* < 0.001; Table 4) in the total cohort. In conclusion, High–PNID status reflects an aggressive disease phenotype and offers accurate prognostic prediction independent of other clinicopathological variables.

**TABLE 2:** Clinicopathological characteristics of patients by PNID status in individual cohorts.

Characteristics	SYMH cohort				Characteristics	SYUTH cohort				Characteristics	KMYAH cohort			
	Non PNID	Low PNID	High PNID	*P*‐value^a^		Non PNID	Low PNID	High PNID	*P*‐value^b^		Non PNID	Low PNID	High PNID	*P*‐value^c^
**N**	164	189	170		**N**	147	76	94		**N**	169	37	45	
**Age, median (IQR), years**	66 (61, 71)	68 (62, 72)	69 (63, 73)	0.055	**Age, mean ± sd, years**	71 ± 8	70 ± 8	69 ± 8	0.341	**Age, mean ± sd, years**	75 ± 8	76 ± 6	74 ± 7	0.547
**BMI, median (IQR), kg/m^2^**	23.42 (21.53, 25.57)	23.73 (21.72, 25.10)	24.19 (22.18, 25.86)	0.102	**BMI, mean ± sd, kg/m^2^**	23.30 ± 2.90	23.37 ± 3.02	24.38 ± 2.95	**0.015**	**BMI, median (IQR), kg/m^2^**	23.44 (21.48, 24.80)	24.66 (20.94, 25.71)	23.95 (21.72, 25.26)	0.241
**tPSA, n (%)**				**0.027**	**tPSA, n (%)**				**< 0.001**	**tPSA, n (%)**				**< 0.001**
< 10 ng/mL	60 (36.6%)	77 (40.7%)	52 (30.6%)		< 10 ng/mL	54 (36.7%)	19 (25.0%)	18 (19.1%)		< 10 ng/mL	71 (42%)	6 (16.2%)	10 (22.2%)	
10‐20 ng/mL	67 (40.9%)	58 (30.7%)	56 (32.9%)		10–20 ng/mL	44 (29.9%)	25 (32.9%)	17 (18.1%)		10‐20 ng/mL	41 (24.3%)	8 (21.6%)	8 (17.8%)	
> 20 ng/mL	37 (22.6%)	54 (28.6%)	62 (36.5%)		> 20 ng/mL	49 (33.3%)	32 (42.1%)	59 (62.8%)		> 20 ng/mL	57 (33.7%)	23 (62.2%)	27 (60.0%)	
**PI‐RADS score, n (%)**				**0.001**	**PI‐RADS score, n (%)**				**< 0.001**	**PI‐RADS score, n (%)**				**< 0.001**
≤ 3	51 (31.1%)	35 (18.5%)	27 (15.9%)		≤ 3	64 (43.5%)	18 (23.7%)	17 (18.1%)		≤ 3	82 (48.5%)	10 (27.0%)	9 (20.0%)	
> 3	113 (68.9%)	154 (81.5%)	143 (84.1%)		> 3	83 (56.5%)	58 (76.3%)	77 (81.9%)		> 3	87 (51.5%)	27 (73.0%)	36 (80.0%)	
**pT, n (%)**				**< 0.001**	**pT, n (%)**				**< 0.001**	**pT, n (%)**				**< 0.001**
1–2	137 (83.5%)	104 (55.0%)	46 (27.1%)		1–2	128 (87.1%)	32 (42.1%)	29 (30.9%)		1–2	137 (81.1%)	15 (40.5%)	18 (40.0%)	
3–4	27 (16.5%)	85 (45.0%)	124 (72.9%)		3–4	19 (12.9%)	44 (57.9%)	65 (69.1%)		3–4	32 (18.9%)	22 (59.5%)	27 (60.0%)	
**pN, n (%)**				**< 0.001**	**pN, n (%)**				**< 0.001**	**pN, n (%)**				**0.008**
0	159 (97%)	169 (89.4%)	121 (71.2%)		0	139 (94.6%)	60 (78.9%)	59 (62.8%)		0	160 (94.7%)	36 (97.3%)	37 (82.2%)	
1	5 (3.0%)	20 (10.6%)	49 (28.8%)		1	8 (5.4%)	16 (21.1%)	35 (37.2%)		1	9 (5.3%)	1 (2.7%)	8 (17.8%)	
**ISUP group, n (%)**				**< 0.001**	**ISUP group, n (%)**				**< 0.001**	**ISUP group, n (%)**				**< 0.001**
1	49 (29.9%)	8 (4.2%)	8 (4.7%)		1	44 (29.9%)	5 (6.6%)	1 (1.1%)		1	44 (26%)	2 (5.4%)	0 (0%)	
2	56 (34.1%)	80 (42.3%)	38 (22.4%)		2	33 (22.4%)	15 (19.7%)	5 (5.3%)		2	57 (33.7%)	9 (24.3%)	6 (13.3%)	
3	26 (15.9%)	49 (25.9%)	48 (28.2%)		3	24 (16.3%)	14 (18.4%)	9 (9.6%)		3	29 (17.2%)	8 (21.6%)	18 (40.0%)	
4	23 (14%)	25 (13.2%)	32 (18.8%)		4	26 (17.7%)	21 (27.6%)	29 (30.9%)		4	23 (13.6%)	10 (27.0%)	10 (22.2%)	
5	10 (6.1%)	27 (14.3%)	44 (25.9%)		5	20 (13.6%)	21 (27.6%)	50 (53.2%)		5	16 (9.5%)	8 (21.6%)	11 (24.4%)	
**PSM, n (%)**				**< 0.001**	**PSM, n (%)**				**< 0.001**	**PSM, n (%)**				**< 0.001**
Negative	119 (72.6%)	108 (57.1%)	52 (30.6%)		Negative	125 (85%)	30 (39.5%)	38 (40.4%)		Negative	126 (74.6%)	17 (45.9%)	25 (55.6%)	
Positive	45 (27.4%)	81 (42.9%)	118 (69.4%)		Positive	22 (15%)	46 (60.5%)	56 (59.6%)		Positive	43 (25.4%)	20 (54.1%)	20 (44.4%)	
**LVI, n (%)**				**< 0.001**	**LVI, n (%)**				**< 0.001**	**LVI, n (%)**				**0.006**
Negative	158 (96.3%)	159 (84.1%)	125 (73.5%)		Negative	141 (95.9%)	59 (77.6%)	67 (71.3%)		Negative	168 (99.4%)	35 (94.6%)	41 (91.1%)	
Positive	6 (3.7%)	30 (15.9%)	45 (26.5%)		Positive	6 (4.1%)	17 (22.4%)	27 (28.7%)		Positive	1 (0.6%)	2 (5.4%)	4 (8.9%)	

*P* values in bold are indicative of statistical significance.

^a^ Kruskal‐Wallis test; Chi‐square test.

^b^ One‐way ANOVA, Chi‐square test.

^c^ One‐way ANOVA; Kruskal‐Wallis test; Chi‐square test. BMI, body mass index; IQR, interquartile range; sd, standard deviation, tPSA, total prostate‐specific antigen; PI‐RADS, Prostate Imaging Reporting and Data System; pT, pathological tumor stage; pN, pathological node stage; ISUP, International Society of Urological Pathology; PSM, positive surgical margin; PNID, perineural invasion density; LVI, lymphovascular invasion; SYMH, Sun Yat‐sen Memorial Hospital; SYUTH, Third Affiliated Hospital of Sun Yat‐sen University; KMYAH, Kunming Medical University Affiliated Yan’ an Hospital.

**TABLE 3:** Time–dependent Cox regression analysis of the association between PNID status and BCR in the Total cohort.

Characteristics	Total	Univariate Analysis			Multivariate Analysis
	HR (95% CI)	*P*‐value		HR (95% CI)	*P*‐value
**PNID**	1091				
Non	480	Reference			Reference
Low	302	1.426 (0.927 –2.192)	0.106	1.036 (0.654 ‐1.641)	0.882
High	309	6.414 (4.615 –8.914)	**< 0.001**	4.056 (2.754 ‐5.973)	**< 0.001**
**Age**	1091	0.966 (0.951 –0.981)	**< 0.001**	0.975 (0.958 ‐0.991)	**0.003**
**BMI**	1091	1.068 (1.021 –1.117)	**0.004**	1.033 (0.989 –1.080)	0.147
**tPSA**	1091				
< 10 ng/mL	367	Reference		Reference	
10‐20 ng/mL	324	1.264 (0.888 –1.798)	0.194	1.108 (0.774–1.585)	0.575
> 20 ng/mL	400	1.710 (1.247 ‐ 2.343)	**< 0.001**	1.002 (0.711–1.412)	0.991
**PI‐RADS score**	1091				
≤ 3	313	Reference			Reference
> 3	778	2.055 (1.478 ‐ 2.858)	**< 0.001**	1.309 (0.929–1.844)	0.123
**pT**	1091				
1‐2	646	Reference			Reference
3‐4	445	2.167 (1.667 –2.817)	**< 0.001**	0.876 (0.635–1.209)	0.420
**pN**	1091				
0	940	Reference			Reference
1	151	2.761 (2.063 –3.696)	**< 0.001**	1.274 (0.911–1.782)	0.157
**ISUP group**	1091				
1	161	Reference			Reference
2	299	1.589 (0.848–2.976)	0.148	1.208 (0.634–2.300)	0.566
3	225	2.622 (1.419 ‐ 4.847)	**0.002**	1.402 (0.728–2.700)	0.312
4	199	3.232 (1.760 ‐ 5.936)	**< 0.001**	1.602 (0.828–3.099)	0.162
5	207	5.479 (3.047 ‐ 9.853)	**< 0.001**	1.998 (1.024–3.897)	**0.042**
**PSM**	1091				
Negative	640	Reference			Reference
Positive	451	2.165 (1.665 ‐ 2.814)	**< 0.001**	1.222 (0.909–1.642)	0.184
**LVI**	1091				
Negative	953	Reference			Reference
Positive	138	3.066 (2.271 ‐ 4.139)	**< 0.001**	1.581 (1.133–2.207)	**0.007**

*P* values in bold are indicative of statistical significance.

HR, Hazard Ratio, CI, Confidence Interval, BMI, body mass index; tPSA, total prostate‐specific antigen; PI‐RADS, Prostate Imaging Reporting and Data System; pT, pathological tumor stage; pN, pathological node stage; ISUP, International Society of Urological Pathology; PSM, positive surgical margin; PNID, perineural invasion density; LVI, lymphovascular invasion.

**TABLE 4:** Time‐dependent Cox regression analysis of the association between PNID status and rPFS in the Total cohort.

Characteristics	Total	Univariate analysis			Multivariate analysis
	HR (95% CI)	*P*‐value		HR (95% CI)	*P*‐value
**PNID**	1091				
Non	480	Reference			Reference
Low	302	1.388 (0.853–2.257)	0.187	1.255 (0.744–2.117)	0.395
High	309	5.800 (4.023–8.360)	**< 0.001**	4.609 (2.967–7.161)	**< 0.001**
**Age**	1091	0.980 (0.963–0.997)	**0.019**	0.985 (0.967–1.003)	0.106
**BMI**	1091	1.043 (0.992–1.098)	0.101		
**tPSA**	1091				
< 10 ng/mL	367	Reference			Reference
10‐20 ng/mL	324	1.408 (0.928–2.134)	0.107	1.266 (0.832–1.925)	0.271
> 20 ng/mL	400	2.049 (1.413–2.973)	**< 0.001**	1.425 (0.956–2.125)	0.082
**PI‐RADS score**	1091				
≤ 3	313	Reference			Reference
> 3	778	1.903 (1.320–2.743)	**< 0.001**	1.186 (0.806–1.746)	0.387
**pT**	1091				
1‐2	646	Reference			Reference
3‐4	445	1.831 (1.366–2.456)	**< 0.001**	0.716 (0.491–1.043)	0.082
**pN**	1091				
0	940	Reference			Reference
1	151	2.752 (1.994–3.798)	**< 0.001**	1.363 (0.933–1.990)	0.109
**ISUP group**	1091				
1	161	Reference			Reference
2	299	1.490 (0.763–2.910)	0.243	1.103 (0.553–2.201)	0.780
3	225	2.523 (1.324–4.810)	**0.005**	1.308 (0.649–2.635)	0.452
4	199	2.579 (1.348–4.935)	**0.004**	1.135 (0.552–2.333)	0.732
5	207	4.450 (2.396–8.264)	**< 0.001**	1.618 (0.790–3.316)	0.188
**PSM**	1091				
Negative	640	Reference			Reference
Positive	451	1.796 (1.340–2.408)	**< 0.001**	0.999 (0.712–1.400)	0.993
**LVI**	1091				
Negative	953	Reference			Reference
Positive	138	2.496 (1.772–3.515)	**< 0.001**	1.273 (0.866–1.873)	0.219

*P* values in bold are indicative of statistical significance. HR, Hazard Ratio, CI, Confidence Interval, BMI, body mass index; tPSA, total prostate‐specific antigen; PI‐RADS, Prostate Imaging Reporting and Data System; pT, pathological tumor stage; pN, pathological node stage; ISUP, International Society of Urological Pathology; PSM, positive surgical margin; PNID, perineural invasion density; LVI, lymphovascular invasion.

Nested Cox proportional hazards models demonstrated that adding PNID to the baseline model improved prognostic performance for BCR (ΔC–index = 0.058, *P* < 0.001; AIC decrease = 91.5 points). For rPFS, the increase in C–index was modest and not statistically significant (ΔC–index = 0.006, *P* = 0.309; AIC decrease = 1.5 points). Complete model comparisons are presented in Table S6.

### 3.3 High–PNID Status Identifies Patients with Potential Therapeutic Benefit from Concurrent β–Blocker Therapy

Prior to therapeutic evaluation, we validated the prognostic significance of PNID in the ADT cohort (n = 309). Kaplan–Meier analysis confirmed that High–PNID status was significantly associated with inferior BCR–free and radiographic progression–free survival (*P* < 0.001 and *P* = 0.002 vs. Non–PNID, respectively; Figure S3). Notably, Low–PNID patients showed outcomes nearly identical to the Non–PNID group (*P* > 0.05 for both endpoints). Based on this prognostic stratification, patients were dichotomized into High–PNID (n = 110) and Non–High PNID (n = 199) for subsequent efficacy analysis. Within the High–PNID subgroup, 30 patients received concurrent β–blockers, 31 received concurrent antimuscarinics, and 61 received ADT alone. In the Non–High PNID cohort, 34 patients received concurrent β–blockers, 65 received concurrent antimuscarinics, and 118 received ADT alone.

In the High–PNID subgroup, concurrent use of β–blockers was associated with a statistically significant reduction in BCR rates. Patients receiving ADT plus β–blockers showed a significantly lower BCR rate compared to ADT alone (16.6% vs. 45.9%; *P* = 0.006; Figure 3A). Survival analysis further supported this trend, with the β–blocker group exhibiting longer BCR–free survival (HR = 0.32, 95% CI: 0.12–0.84; *P* = 0.021; Figure 3C). In the Non–High PNID subgroup, concurrent use of β–blockers showed no such association. BCR rates were comparable to controls (14.7% vs. 14.4%; *P* = 1.000; Figure 3B), and no significant difference was observed in BCR–free survival (HR = 0.62, 95% CI: 0.22–1.75; *P* = 0.363; Figure 3D). Regarding antimuscarinic agents, no significant therapeutic benefits were observed in either the High–PNID or Non–High PNID subgroups for BCR rates or BCR–free survival compared to controls (*P* > 0.05 for all; Figure S4).

**FIGURE 3:**
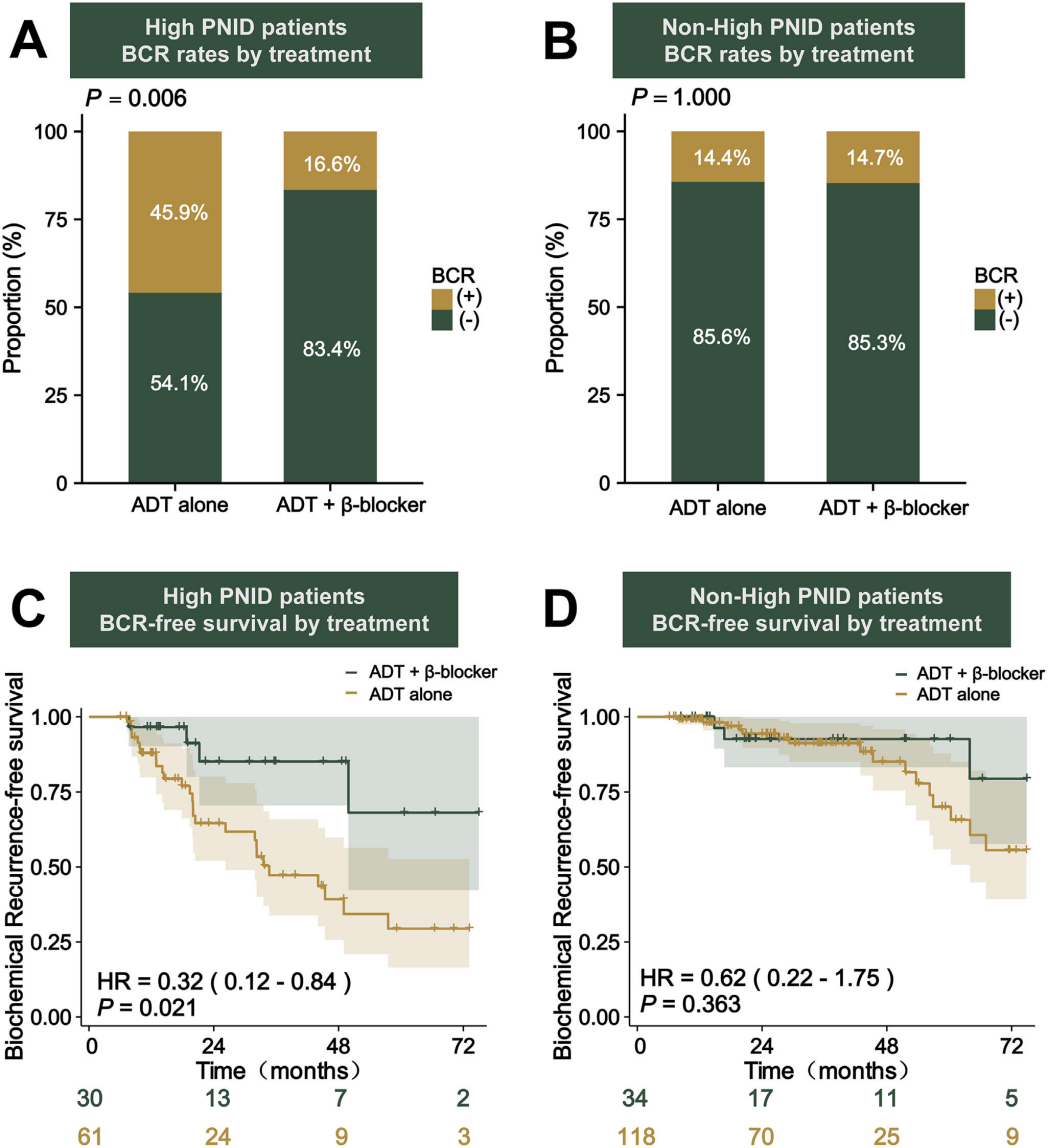
Therapeutic impact of concurrent β‐blockers stratified by PNID status. (A) Comparison of BCR rates between treatment groups in the High‐PNID subgroup. (B) Comparison of BCR rates between treatment groups in the Non‐High PNID subgroup. (C) Kaplan–Meier curves for BCR‐free survival between treatment groups in the High‐PNID subgroup. (D) Kaplan‐Meier curves for BCR‐free survival between treatment groups in the Non‐High PNID subgroup. ADT, androgen deprivation therapy; BCR, biochemical recurrence; PNID, perineural invasion density.

### 3.4 The AI Model Accurately Predicts High–PNID and Stratifies Patient Prognosis

Patients from SYMH (n = 523) were randomly divided into a training cohort (n = 314) and an internal validation cohort (n = 209) at a 6:4 ratio, while patients from SYUTH (n = 317) and KMYAH (n = 251) constituted external validation cohorts 1 and 2, respectively, to assess external generalizability. Given the substantial prognostic significance of High–PNID status and the clinical necessity for its non–invasive assessment, we developed an AI model utilizing MR images to identify High–PNID patients from Non–High PNID patients. Figure 4A presents representative examples of the workflow, including the original MR image, tumor mask, T2WI–ROI, Guided Grad–CAM heatmap, and the corresponding H &E assessment.

**FIGURE 4:**
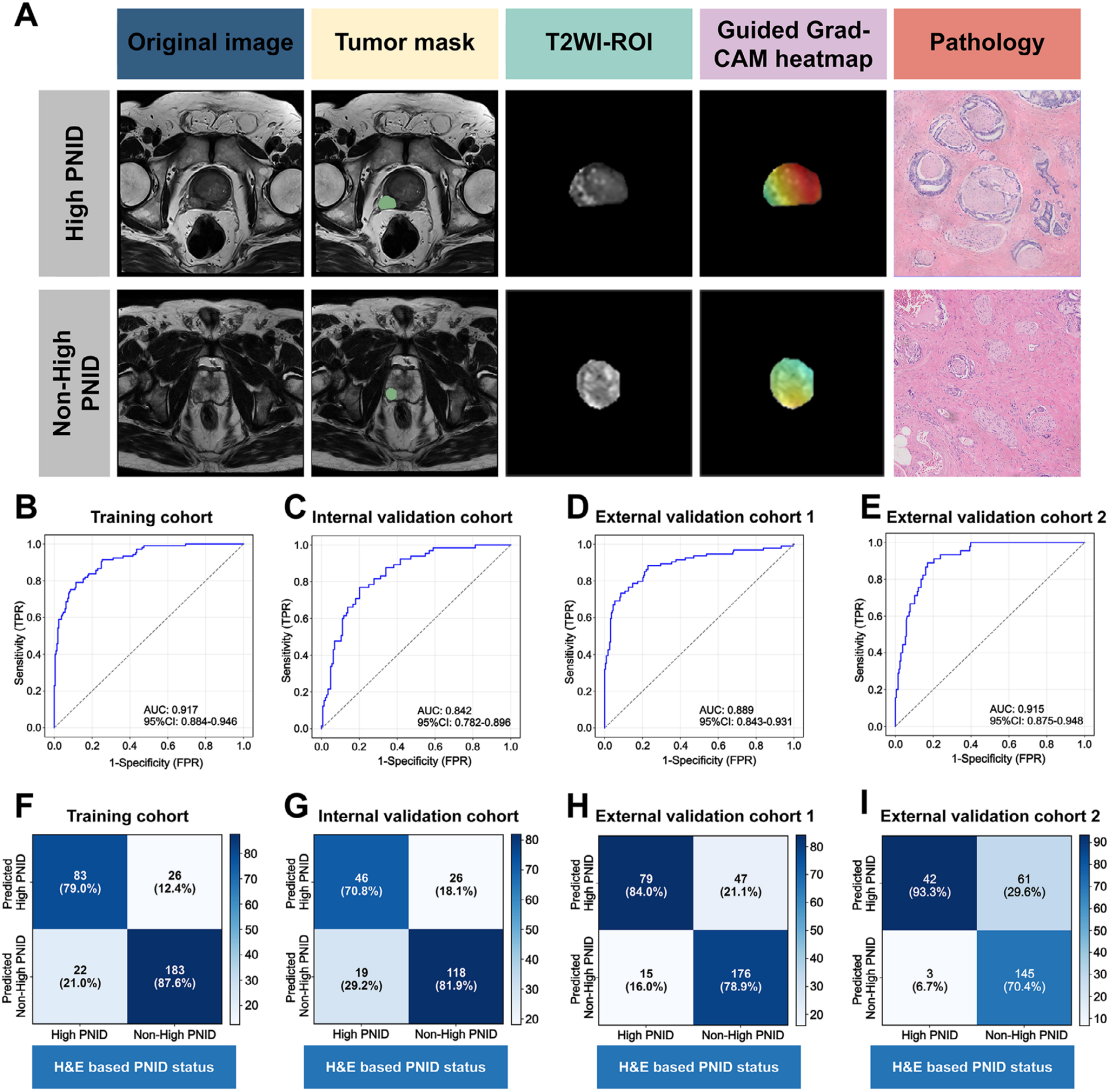
Performance of the PNID based artificial intelligence prediction model across different cohorts. (A) Representative examples of the original MR images, tumor mask and T2WI ROI. The model prediction is visualized using Guided Grad‐CAM, along with the corresponding pathological images. (B–E) AUCs and 95% CIs of results obtained using the artificial intelligence PNID prediction model for the training cohort (n = 314), internal validation cohort (n = 209), external validation cohort 1 (n = 317), and external validation cohort 2 (n = 251). (F–I) Sensitivity and specificity of results obtained using the PNID based artificial intelligence prediction model for the training cohort, internal validation cohort, external validation cohort 1, and external validation cohort 2. AUC, the area under the curve; 95% CI, 95% confidence interval; MR, magnetic resonance; Guided Grad‐CAM, guided gradient‐weighted class activation mapping; H&E, hematoxylin and eosin; ROI, region of interest; PNID, perineural invasion density.

In the training cohort, the model demonstrated high diagnostic performance, achieving an AUC of 0.917 (95% CI: 0.884–0.946) for predicting High–PNID status (Figure 4B). The model exhibited robust generalizability across validation datasets, yielding AUCs of 0.842 (95% CI: 0.782–0.896), 0.889 (95% CI: 0.843–0.931), and 0.915 (95% CI: 0.875–0.948) for the internal validation cohort, external validation cohort 1, and external validation cohort 2, respectively (Figure 4C–E). The overall prediction accuracies were 84.7%, 78.5%, 80.4%, and 74.5% for the training, internal, and two external validation cohorts, respectively. Comprehensive performance metrics, including sensitivity, specificity, PPV, and NPV, are detailed in Figure 4F–I and Table 5, underscoring the model's robust discriminative power across all cohorts.

**TABLE 5:** Performance of the PNID‐based deep learning prediction model in the training and validation cohorts.

	AUC (95% CI)	Sensitivity (95% CI)	Specificity (95% Cl)	PPV (95% Cl)	NPV (95% Cl)	Overall accuracy
**Training cohort**	0.917	0.790	0.876	0.761	0.893	84.70%
	(0.884–0.946)	(0.703–0.857)	(0.824–0.914)	(0.673–0.832)	(0.843–0.928)	
**Internal validation cohort**	0.842	0.708	0.819	0.639	0.861	78.50%
	(0.782–0.896)	(0.588–0.804)	(0.749–0.874)	(0.524–0.740)	(0.794–0.909)	
**External validation cohort 1**	0.889	0.840	0.789	0.627	0.921	80.40%
	(0.843–0.931)	(0.753–0.901)	(0.731–0.838)	(0.540–0.706)	(0.874–0.952)	
**External validation cohort 2**	0.915	0.933	0.704	0.408	0.980	74.50%
	(0.875–0.948)	(0.821–0.977)	(0.638–0.762)	(0.318–0.504)	(0.942–0.993)	

AUC, area under the curve; 95% CI, 95% confidence interval; NPV, negative predictive value; PPV, positive predictive value; PNID, perineural invasion density.

Beyond diagnostic accuracy, the model demonstrated significant prognostic stratification capability. Predicted High–PNID patients experienced significantly worse BCR across all cohorts (all *P* < 0.05; Figure 5A–D) and rapid radiographic progression in the training and external cohorts (all *P* < 0.05; Figure 5E,G,H). Although a numerical trend toward rPFS separation was observed in the internal validation cohort (HR: 1.43), this did not reach statistical significance (*P* = 0.296; Figure 5F), potentially due to the limited number of progression events. Multivariate Cox regression confirmed the model's independent predictive value in overall cohorts (Tables S4 and S5). As shown in Figure S5, an optimized probability threshold of 0.35 effectively stratified 37.6% of the population into the high–risk group, showing high concordance with histological High–PNID status.

**FIGURE 5:**
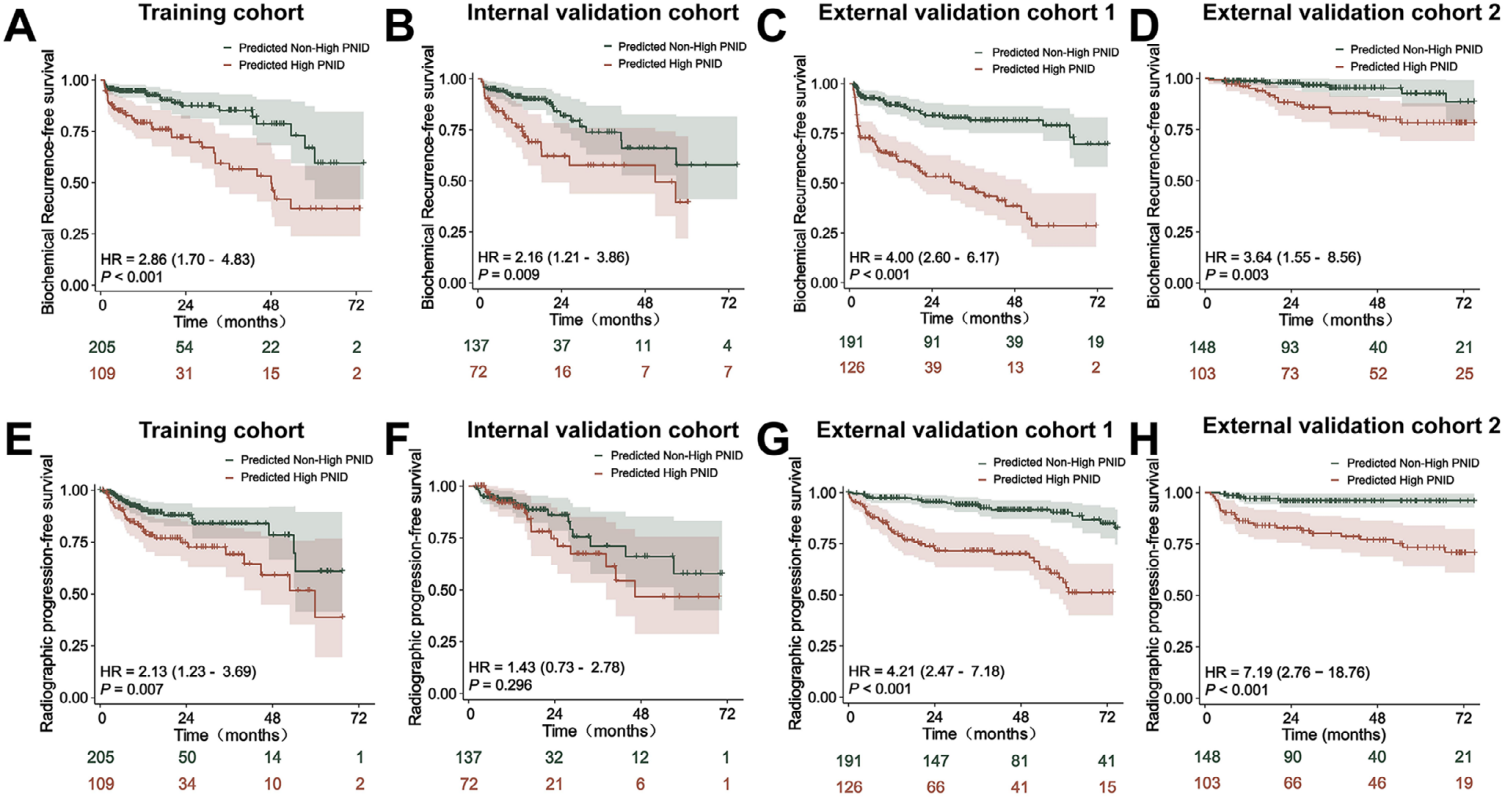
Evaluation of the model's prognostic value. (A–D) Kaplan–Meier analysis of the BCR survival of patients with PCa stratified by the predicted PNID status in the training cohort, internal validation cohort, external validation cohort 1, and external validation cohort 2. (E–H) Kaplan–Meier analysis of the rPFS of patients with PCa stratified by the predicted PNID status in the training cohort, internal validation cohort, external validation cohort 1, and external validation cohort 2. *P* values were calculated using the Cox proportional hazards model. PNID, perineural invasion density; HR, hazard ratio.

## 4 Discussion

In this multicenter cohort study, we established a quantitative metric, termed PNID, and demonstrated the robust prognostic value of High–PNID status in PCa. Subsequently, our subgroup analysis suggested a potential benefit of combining β–blockers with adjuvant ADT specifically for High–PNID patients. Finally, we developed and validated an AI–based model to non–invasively assess High–PNID status using MR images.

PNI has been established as a critical histopathological feature associated with aggressive tumor behavior and poor prognosis across multiple malignancies, such as pancreatic cancer and colorectal cancer [24, 28, 29]. However, the prognostic value of PNI remains controversial in PCa. This discrepancy likely arises because conventional qualitative assessments fail to capture the density–dependent heterogeneity of neural involvement, particularly given the abundance of nerve fibers in the prostate. While recent studies have demonstrated the prognostic value of quantifying PNI foci [11, 12, 13], they rely on absolute counts that ignore the background density of non–invaded nerves and employ inconsistent definitions of the measurement region. By introducing PNID, we shift the paradigm from absolute counting to a proportional assessment. Calculating the ratio of invaded to total nerves at the invasive front normalizes for local nerve distribution, providing a more biologically accurate measure of the tumor's intrinsic invasive potential. Notably, our multicenter analysis established High PNID status as an independent risk factor for BCR and rPFS, with incremental prognostic value beyond existing pathological factors included in the baseline model for BCR prediction.

From a biological perspective, established literature in cancer neuroscience suggests that PNI reflects a dynamic bidirectional crosstalk between tumor cells and nerves. On one hand, tumor cells secrete neurotrophic signaling molecules to facilitate nerve recruitment and tumor migration along neural tracts [30]. On the other hand, autonomic nerve fibers release neurotransmitters such as norepinephrine and acetylcholine, thereby orchestrating cancer initiation, survival, and dissemination by activating receptor–mediated angiogenic and metabolic switches within the tumor microenvironment [2, 3, 31]. This nerve–tumor co–dependency facilitates metastatic spread and establishes a self–reinforcing oncogenic loop. Targeting the nerve–oncology axis has shown therapeutic promise; denervation inhibits PCa development in mouse models [2], while clinical data suggest that β–blockers may reduce recurrence and delay castration resistance [25, 32]. Autonomic innervation in PCa is predominantly characterized by sympathetic adrenergic fibers that fuel tumor progression [2]. In this context, High PNID morphologically reflects an extensive nerve–tumor contact surface, which may plausibly expose residual cancer cells to heightened perineural adrenergic stimulation. Under systemic androgen ablation, adrenergic stress signaling has been documented to potentially serve as a compensatory survival mechanism that counteracts apoptosis via the ADRB2/cAMP/PKA/BAD pathway in preclinical models [33, 34]. Consequently, we hypothesize that High PNID tumors might be relatively more reliant on this microenvironmental adrenergic support, rendering them potentially susceptible to concurrent. Our subgroup analysis of patients provided preliminary clinical support for this hypothesis. Specifically, the High PNID patients treated with concurrent β–blockers exhibited higher rates of freedom from BCR compared with those receiving ADT alone. This differential treatment response suggests that β–blockers may confer a more pronounced therapeutic benefit in High PNID patients, a benefit not observed in Non–High PNID patients.

Interestingly, concurrent antimuscarinic therapy yielded no comparable survival benefit. This divergence likely stems from both biological and clinical distinctions. Biologically, while parasympathetic muscarinic signaling predominantly mediates local motility and invasion during early tumorigenesis, sympathetic adrenergic signaling sustains residual micrometastasis survival and angiogenic remodeling under castration pressure [2, 3, 33]. Clinically, β–blockers involve continuous, long–term maintenance for chronic cardiovascular comorbidities, whereas antimuscarinics are typically prescribed on a transient, on–demand basis for postoperative lower urinary tract symptoms [35, 36]. Consequently, brief muscarinic inhibition appears insufficient to achieve therapeutic microenvironmental remodeling in post–prostatectomy patients undergoing ADT. These findings provide exploratory clinical evidence for nerve–targeted adjuvant strategies and should be validated in larger prospective cohorts.

However, current assessments of PNID rely on postoperative pathological H &E–stained slides, which poses two major challenges for clinical implementation. First, manual quantification is labor–intensive and inefficient. Second, PNID status can only be obtained postoperatively. Given the significant prognostic value of PNID and its potential to guide risk stratification for adjuvant therapy, there is an urgent need for the non–invasive prediction of PNID. To address this challenge, AI–driven image analysis has emerged as a powerful tool for non–invasive tumor characterization [20, 37, 38, 39]. In light of this, we constructed and validated a deep learning convolutional neural network (CNN) model based on MRI for the preoperative prediction of PNID status. Specifically, we utilized the ResNet–50 framework, which employs residual blocks to mitigate gradient degradation and capture complex features, thereby ensuring robust generalization and high prediction accuracy [40, 41]. To the best of our knowledge, this is a pioneering effort to leverage AI for identifying this specific high–risk pathological feature in PCa. Our model not only demonstrates robust generalization across cohorts as a reliable non–invasive alternative, but also grounds its predictions in the specific pathological context of PNID, thereby ensuring that high diagnostic accuracy is supported by clear clinical interpretability.

Our study has several limitations. First, the retrospective design inherently introduces selection bias and confounding factors. Therefore, validation in large–scale prospective cohorts is essential. Second, although rigorous inclusion and exclusion criteria were applied in therapy subgroup, the adjuvant therapy regimens could not be completely standardized. To address this, we are planning a Phase Ib clinical trial to further validate our findings (ChiCTR2600131959). Third, although evaluating PNID within a standardized hotspot at the deepest invasive boundary achieves acceptable inter–observer reproducibility, this focal sampling approach cannot fully capture the spatial heterogeneity of nerve distribution across the entire prostate specimen. Similarly, tumor ROI delineation currently relies on manual segmentation. Future investigations integrating whole–slide digital pathology with automated image analysis will enable comprehensive neural and tumor quantification while improving reproducibility.

In summary, we established PNID as a superior prognostic biomarker compared to conventional PNI. This approach facilitates precise risk stratification and suggests that High–PNID patients may specifically benefit from concurrent β–blocker therapy. Our deep learning model, developed using preoperative MR images, enables the accurate, non–invasive prediction of High–PNID status in patients with PCa. Prospective clinical trials are warranted to validate these findings and translate this nerve–targeted strategy into clinical practice.

## Author Contributions

B. Liu, W. Zhong, and Y. Luo designed the study. B. Liu, L. Zhang, W. Zhu, M. Yang, X. Liu, and X. Li integrated and analyzed the data. B. Liu, X. Li, P. Qin, Z. Lin, Q. Cai, J. Zheng, X. Zhao, C. Zou, H. Su, D. Wu, L. Xu, L. Zhang, and C. Zhang collected the clinical data and performed the analysis. Y. Luo, C. Wang, W. Zhong, K. Li and X. Liu obtained funding. B. Liu prepared the figures. W. Zhong and Y. Luo revised the manuscript and supervised the study. All authors had full access to all the data, were involved in drafting the manuscript, and read and approved the final manuscript.

## Conflicts of Interest

The authors declare no conflicts of interest.

## Supporting information




**Supporting File**: advs78460‐sup‐0001‐SuppMat.docx.

## Data Availability

The data that support the findings of this study are available from the corresponding author upon reasonable request.
