# Supplementary material for: Non‐Invasive Assessment of Perineural Invasion Density: A Novel Marker for Prognosis and Treatment Stratification in Prostate Cancer
Source: Adv Sci (Weinh). 2026 Oct 31:e78460. Online ahead of print. doi: 10.1002/advs.78460 (PMC13723708; doi:10.1002/advs.78460)
Supplement: Supplementary file 1 [file ADVS-9999-e78460-s001.docx]

**Supplementary Material for**

**Non-Invasive Assessment of Perineural Invasion Density: A Novel Marker for Prognosis and Treatment Stratification in Prostate Cancer**

**Supplementary Methods**

Supplementary 1. Multiplex immunofluorescence staining

Supplementary 2. Statistical Methods for Incremental Prognostic Value Assessment

**Supplementary Figures and Tables**

Supplementary Figure 1. Study flowchart.

Supplementary Figure 2. Prognostic value of the PNI status in all cohorts.

Supplementary Figure 3. Prognostic validation of PNID status in the ADT cohort.

Supplementary Figure 4. Therapeutic impact of concurrent antimuscarinics stratified by PNID status.

Supplementary Figure 5. Distribution of predicted risk scores and alignment with actual PNID status.

Supplementary Figure 6. Multiplex immunofluorescence staining of the PNID subgroups.

Supplementary Table 1. Detailed classification of pharmacological agents included in the analysis.

Supplementary Table 2. Time-dependent Cox regression analysis of the association between PNI and BCR in the Total cohort.

Supplementary Table 3. Time-dependent Cox regression analysis of the association between PNI and rPFS in the Total cohort.

Supplementary Table 4. Time-dependent Cox regression analysis of the association between predicted PNID status and BCR in the Total cohort.

Supplementary Table 5. Time-dependent Cox regression analysis of the association between predicted PNID status and rPFS in the Total cohort.

Supplementary Table 6. Incremental Prognostic Value of PNID via Nested Cox Models

**Supplementary 1**. Multiplex immunofluorescence staining

Multiplex immunofluorescence analysis was performed on formalin-fixed, paraffin-embedded tissue sections. Following deparaffinization, rehydration, and heat-induced epitope retrieval, endogenous peroxidase activity was quenched and nonspecific binding was blocked. Sequential immunostaining cycles were conducted using primary antibodies against S100 and Pan-CK, with each cycle comprising primary antibody incubation, horseradish peroxidase-conjugated secondary antibody application, and tyramide signal amplification. Antibody stripping via heat-mediated antigen retrieval between cycles prevented cross-reactivity. Nuclear counterstaining was performed with DAPI, and multispectral images were acquired using an automated quantitative pathology imaging system (Akoya Vectra Polaris). Image selection and quantitative analysis were conducted blinded to PNID status.

**Supplementary Methods 2.** Statistical Methods for Incremental Prognostic Value Assessment

Nested Cox proportional hazards models were constructed with three sequential steps: Model 1 included baseline pathological factors (tPSA category, PI-RADS, pathological T stage, pathological N stage, ISUP grade group, positive surgical margin, and lymphovascular invasion); Model 2 added qualitative PNI status; Model 3 added binary High-PNID status (≥50% vs. <50%). Harrell’s concordance index (C-index) was calculated for each model with 95% confidence intervals estimated via paired bootstrap resampling (300 iterations). Incremental prognostic value was assessed using likelihood ratio tests and Akaike Information Criterion (AIC).

**Supplementary Figure 1. Study flowchart.**

PCa, prostate cancer; MRI, magnetic resonance imaging; H&E, haematoxylin and eosin; ADT, androgen deprivation therapy; AI, artificial intelligence; PNID, perineural invasion density; SYMH, Sun Yat-sen Memorial Hospital; SYUTH, Third Affiliated Hospital of Sun Yat-sen University; KMYAH, Kunming Medical University Affiliated Yan’an Hospital.

**Supplementary Figure 2. Prognostic value of the PNI status in all cohorts.**

**A-F)** Kaplan-Meier analysis of BCR and rPFS of patients with PCa stratified by the PNI status in the SYMH, SYUTH and KMYAH cohorts.

PNI, perineural invasion; BCR, Biochemical recurrence; rPFS, Radiographic progression‑free survival; *P*-values were calculated using the Cox proportional hazards model. SYMH, Sun Yat-sen Memorial Hospital; SYUTH, Third Affiliated Hospital of Sun Yat-sen University; KMYAH, Kunming Medical University Affiliated Yan’ an Hospital; HR, hazard ratio.

**Supplementary Figure 3**. Prognostic validation of PNID status in the ADT cohort.

**A)** Kaplan-Meier curves for BCR-free survival stratified by PNID status in the ADT cohort.
**B**) Kaplan-Meier curves for rPFS-free survival stratified by PNID status in the ADT cohort.
ADT, androgen deprivation therapy; PNID, perineural invasion density.

**Supplementary Figure 4**. Therapeutic impact of concurrent antimuscarinics stratified by PNID status.

**A**) Comparison of BCR rates between treatment groups in the High-PNID subgroup.
**B**) Comparison of BCR rates between treatment groups in the Non-High PNID subgroup.
**C**) Kaplan-Meier curves for BCR-free survival between treatment groups in the High-PNID subgroup.
**D**) Kaplan-Meier curves for BCR-free survival between treatment groups in the Non-High PNID subgroup.
ADT, androgen deprivation therapy; BCR, biochemical recurrence; PNID, perineural invasion density.

**Supplementary Figure 5. Distribution of predicted risk scores and alignment with actual PNID status.**

Composite chart depicting the relationship between PNID prediction status and actual results, along with a risk stratification system based on a selected threshold of 0.35.

PNID, perineural invasion density.

**Supplementary Figure 6. Multiplex immunofluorescence staining of the PNID subgroups.**

Representative multiplex immunofluorescence staining of radical prostatectomy specimens across High-PNID (top row), Low-PNID (middle row), and Non-PNID (bottom row) groups. Columns from left to right display merged composite images, Pan-CK+ prostate cancer epithelial cells (yellow) with DAPI-stained nuclei (blue), and S100+ nerve bundles (orange) with DAPI-stained nuclei (blue). Scale bars = 100μm. Abbreviations: PNID, perineural invasion density; Pan-CK, pan-cytokeratin; DAPI, 4',6-diamidino-2-phenylindole.

| **Table S1. Detailed classification of pharmacological agents included in the analysis.** | | | |
| --- | --- | --- | --- |
| **Drug Class** | **Pharmacological Sub-classification** | **Generic Name** | **WHO ATC Code** |
| **LHRH Agonists** | GnRH analogues | Leuprorelin | L02AE02 |
|  | GnRH analogues | Goserelin | L02AE03 |
|  | GnRH analogues | Triptorelin | L02AE04 |
| **AR Inhibitors** | First-generation antiandrogens | Bicalutamide | L02BB03 |
|  | Next-generation antiandrogens | Enzalutamide | L02BB04 |
|  | Next-generation antiandrogens | Apalutamide | L02BB05 |
|  | Next-generation antiandrogens | Darolutamide | L02BB06 |
| **β-blockers** | Selective β-blocking agents | Bisoprolol | C07AB07 |
|  | Selective β-blocking agents | Metoprolol | C07AB02 |
|  | Non-selective β-blocking agents | Propranolol | C07AA05 |
| **Antimuscarinics** | Muscarinic antagonists | Solifenacin | G04BD08 |
|  | Muscarinic antagonists | Tolterodine | G04BD07 |
| LHRH, luteinizing hormone–releasing hormone; GnRH, gonadotropin-releasing hormone; AR, androgen receptor; WHO, World Health Organization; ATC, Anatomical Therapeutic Chemical. | | | |

| **Table S2. Time-dependent Cox regression analysis of the association between PNI and BCR in the Total cohort.** | | | | | | |
| --- | --- | --- | --- | --- | --- | --- |
| **Characteristics** | **Total** | **Univariate analysis** | |  | **Multivariate analysis** | |
|  |  | **HR (95% CI)** | P-value |  | **HR (95% CI)** | P-value |
| **PNI** | 1091 |  |  |  |  |  |
| Negative | 480 | Reference |  |  | Reference |  |
| Positive | 611 | 3.730 (2.708 - 5.138) | **< 0.001** |  | 2.307 (1.598 - 3.330) | **< 0.001** |
| **Age** | 1091 | 0.966 (0.951 - 0.981) | **< 0.001** |  | 0.974 (0.958 - 0.991) | **0.002** |
| **BMI** | 1091 | 1.068 (1.021 - 1.117) | **0.004** |  | 1.041 (0.996 - 1.089) | 0.076 |
| **tPSA** | 1091 |  |  |  |  |  |
| < 10 ng/mL | 367 | Reference |  |  | Reference |  |
| 10-20 ng/mL | 324 | 1.264 (0.888 - 1.798) | 0.194 |  | 1.153 (0.806 - 1.649) | 0.436 |
| > 20 ng/mL | 400 | 1.710 (1.247 - 2.343) | **< 0.001** |  | 1.040 (0.739 - 1.462) | 0.823 |
| **PI-RADS score** | 1091 |  |  |  |  |  |
| ≤ 3 | 313 | Reference |  |  | Reference |  |
| > 3 | 778 | 2.055 (1.478 - 2.858) | **< 0.001** |  | 1.286 (0.915 - 1.808) | 0.148 |
| **pT** | 1091 |  |  |  |  |  |
| 1-2 | 646 | Reference |  |  | Reference |  |
| 3-4 | 445 | 2.167 (1.667 - 2.817) | **< 0.001** |  | 0.965 (0.701 - 1.328) | 0.827 |
| **pN** | 1091 |  |  |  |  |  |
| 0 | 940 | Reference |  |  | Reference |  |
| 1 | 151 | 2.761 (2.063 - 3.696) | **< 0.001** |  | 1.457 (1.045 - 2.032) | **0.027** |
| **ISUP group** | 1091 |  |  |  |  |  |
| 1 | 161 | Reference |  |  | Reference |  |
| 2 | 299 | 1.589 (0.848 - 2.976) | 0.148 |  | 1.019 (0.533 - 1.949) | 0.954 |
| 3 | 225 | 2.622 (1.419 - 4.847) | **0.002** |  | 1.471 (0.767 - 2.823) | 0.246 |
| 4 | 199 | 3.232 (1.760 - 5.936) | **< 0.001** |  | 1.622 (0.837 - 3.142) | 0.152 |
| 5 | 207 | 5.479 (3.047 - 9.853) | **< 0.001** |  | 2.152 (1.107 - 4.184) | **0.024** |
| **PSM** | 1091 |  |  |  |  |  |
| Negative | 640 | Reference |  |  | Reference |  |
| Positive | 451 | 2.165 (1.665 - 2.814) | **< 0.001** |  | 1.219 (0.904 - 1.643) | 0.194 |
| **LVI** | 1091 |  |  |  |  |  |
| Negative | 953 | Reference |  |  | Reference |  |
| Positive | 138 | 3.066 (2.271 - 4.139) | **< 0.001** |  | 1.497 (1.074 - 2.087) | **0.017** |
| *P* values in bold are indicative of statistical significance. HR, Hazard Ratio; CI, Confidence Interval, BMI, body mass index; tPSA, total prostate-specific antigen; PI-RADS, Prostate Imaging Reporting and Data System; pT, pathological tumor stage; pN, pathological node stage; ISUP, International Society of Urological Pathology; PSM, positive surgical margin; PNI, perineural invasion; LVI, lymphovascular invasion. | | | | | | |

| **Table S3. Time-dependent Cox regression analysis of the association between PNI and rPFS in the Total cohort.** | | | | | | |
| --- | --- | --- | --- | --- | --- | --- |
| **Characteristics** | **Total** | **Univariate analysis** | |  | **Multivariate analysis** | |
|  |  | **HR (95% CI)** | ***P*-value** |  | **HR (95% CI)** | ***P*-value** |
| **PNI** | 1091 |  |  |  |  |  |
| Negative | 480 | Reference |  |  | Reference |  |
| Positive | 611 | 1.354 (1.005 - 1.825) | **0.046** |  | 1.177 (0.864 - 1.603) | 0.302 |
| **Age** | 1091 | 0.980 (0.963 - 0.997) | **0.019** |  | 0.981 (0.964 - 0.999) | **0.036** |
| **BMI** | 1091 | 1.043 (0.992 - 1.098) | 0.101 |  |  |  |
| **tPSA** | 1091 |  |  |  |  |  |
| < 10 ng/mL | 367 | Reference |  |  | Reference |  |
| 10-20 ng/mL | 324 | 1.408 (0.928 - 2.134) | 0.107 |  | 1.324 (0.870 - 2.016) | 0.190 |
| > 20 ng/mL | 400 | 2.049 (1.413 - 2.973) | **< 0.001** |  | 1.419 (0.958 - 2.103) | 0.081 |
| **PI-RADS score** | 1091 |  |  |  |  |  |
| ≤ 3 | 313 | Reference |  |  | Reference |  |
| > 3 | 778 | 1.903 (1.320 - 2.743) | **< 0.001** |  | 1.299 (0.884 - 1.910) | 0.183 |
| **pT** | 1091 |  |  |  |  |  |
| 1-2 | 646 | Reference |  |  | Reference |  |
| 3-4 | 445 | 1.831 (1.366 - 2.456) | **< 0.001** |  | 0.924 (0.636 - 1.340) | 0.676 |
| **pN** | 1091 |  |  |  |  |  |
| 0 | 940 | Reference |  |  | Reference |  |
| 1 | 151 | 2.752 (1.994 - 3.798) | **< 0.001** |  | 1.653 (1.131 - 2.417) | **0.009** |
| **ISUP group** | 1091 |  |  |  |  |  |
| 1 | 161 | Reference |  |  | Reference |  |
| 2 | 299 | 1.490 (0.763 - 2.910) | 0.243 |  | 1.301 (0.660 - 2.566) | 0.447 |
| 3 | 225 | 2.523 (1.324 - 4.810) | **0.005** |  | 1.895 (0.962 - 3.729) | 0.064 |
| 4 | 199 | 2.579 (1.348 - 4.935) | **0.004** |  | 1.783 (0.890 - 3.575) | 0.103 |
| 5 | 207 | 4.450 (2.396 - 8.264) | **< 0.001** |  | 2.489 (1.243 - 4.984) | **0.010** |
| **PSM** | 1091 |  |  |  |  |  |
| Negative | 640 | Reference |  |  | Reference |  |
| Positive | 451 | 1.796 (1.340 - 2.408) | **< 0.001** |  | 1.157 (0.824 - 1.625) | 0.399 |
| **LVI** | 1091 |  |  |  |  |  |
| Negative | 953 | Reference |  |  | Reference |  |
| Positive | 138 | 2.496 (1.772 - 3.515) | **< 0.001** |  | 1.453 (0.992 - 2.129) | 0.055 |
| *P* values in bold are indicative of statistical significance. HR, Hazard Ratio; CI, Confidence Interval, BMI, body mass index; tPSA, total prostate-specific antigen; PI-RADS, Prostate Imaging Reporting and Data System; pT, pathological tumor stage; pN, pathological node stage; ISUP, International Society of Urological Pathology; PSM, positive surgical margin; PNI, perineural invasion; LVI, lymphovascular invasion. | | | | | | |

| **Table S4. Time-dependent Cox regression analysis of the association between predicted PNID status and BCR in the Total cohort.** | | | | | | |
| --- | --- | --- | --- | --- | --- | --- |
| **Characteristics** | **Total** | **Univariate analysis** | |  | **Multivariate analysis** | |
|  |  | **HR (95% CI)** | ***P*-value** |  | **HR (95% CI)** | ***P*-value** |
| **Predicted PNID** | 1091 |  |  |  |  |  |
| Non-High | 681 | Reference |  |  | Reference |  |
| High | 410 | 2.857 (2.185 - 3.735) | **< 0.001** |  | 2.389 (1.803 - 3.165) | **< 0.001** |
| **Age** | 1091 | 0.966 (0.951 - 0.981) | **< 0.001** |  | 0.962 (0.946 - 0.978) | **< 0.001** |
| **BMI** | 1091 | 1.068 (1.021 - 1.117) | **0.004** |  | 1.030 (0.985 - 1.076) | 0.199 |
| **tPSA** | 1091 |  |  |  |  |  |
| < 10 ng/mL | 367 | Reference |  |  | Reference |  |
| 10-20 ng/mL | 324 | 1.264 (0.888 - 1.798) | 0.194 |  | 1.185 (0.829 - 1.695) | 0.352 |
| > 20 ng/mL | 400 | 1.710 (1.247 - 2.343) | **< 0.001** |  | 1.046 (0.748 - 1.462) | 0.795 |
| **PI-RADS score** | 1091 |  |  |  |  |  |
| ≤ 3 | 313 | Reference |  |  | Reference |  |
| > 3 | 778 | 2.055 (1.478 - 2.858) | **< 0.001** |  | 1.328 (0.942 - 1.873) | 0.105 |
| **pT** | 1091 |  |  |  |  |  |
| 1-2 | 646 | Reference |  |  | Reference |  |
| 3-4 | 445 | 2.167 (1.667 - 2.817) | **< 0.001** |  | 1.066 (0.775 - 1.465) | 0.696 |
| **pN** | 1091 |  |  |  |  |  |
| 0 | 940 | Reference |  |  | Reference |  |
| 1 | 151 | 2.761 (2.063 - 3.696) | **< 0.001** |  | 1.430 (1.027 - 1.993) | **0.034** |
| **ISUP group** | 1091 |  |  |  |  |  |
| 1 | 161 | Reference |  |  | Reference |  |
| 2 | 299 | 1.589 (0.848 - 2.976) | 0.148 |  | 1.211 (0.640 - 2.290) | 0.556 |
| 3 | 225 | 2.622 (1.419 - 4.847) | **0.002** |  | 1.691 (0.889 - 3.219) | 0.109 |
| 4 | 199 | 3.232 (1.760 - 5.936) | **< 0.001** |  | 1.895 (0.988 - 3.634) | 0.054 |
| 5 | 207 | 5.479 (3.047 - 9.853) | **< 0.001** |  | 2.269 (1.172 - 4.392) | **0.015** |
| **PSM** | 1091 |  |  |  |  |  |
| Negative | 640 | Reference |  |  | Reference |  |
| Positive | 451 | 2.165 (1.665 - 2.814) | **< 0.001** |  | 1.345 (0.998 - 1.813) | 0.052 |
| **LVI** | 1091 |  |  |  |  |  |
| Negative | 953 | Reference |  |  | Reference |  |
| Positive | 138 | 3.066 (2.271 - 4.139) | **< 0.001** |  | 1.586 (1.134 - 2.218) | **0.007** |
| *P* values in bold are indicative of statistical significance. HR, Hazard Ratio; CI, Confidence Interval, BMI, body mass index; tPSA, total prostate-specific antigen; PI-RADS, Prostate Imaging Reporting and Data System; pT, pathological tumor stage; pN, pathological node stage; ISUP, International Society of Urological Pathology; PSM, positive surgical margin; PNID, perineural invasion density; LVI, lymphovascular invasion. | | | | | | |

| **Table S5. Time-dependent Cox regression analysis of the association between Predicted PNID status and rPFS in the Total cohort.** | | | | | | |
| --- | --- | --- | --- | --- | --- | --- |
| **Characteristics** | **Total** | **Univariate analysis** | |  | **Multivariate analysis** | |
|  |  | **HR (95% CI)** | ***P*-value** |  | **HR (95% CI)** | ***P*-value** |
| **Predicted PNID** | 1091 |  |  |  |  |  |
| Non-High | 681 | Reference |  |  | Reference |  |
| High | 410 | 2.841 (2.101 - 3.842) | **< 0.001** |  | 2.345 (1.698 - 3.238) | **< 0.001** |
| **Age** | 1091 | 0.980 (0.963 - 0.997) | **0.019** |  | 0.973 (0.955 - 0.991) | **0.003** |
| **BMI** | 1091 | 1.043 (0.992 - 1.098) | 0.101 |  |  |  |
| **tPSA** | 1091 |  |  |  |  |  |
| < 10 ng/mL | 367 | Reference |  |  | Reference |  |
| 10-20 ng/mL | 324 | 1.408 (0.928 - 2.134) | 0.107 |  | 1.331 (0.875 - 2.026) | 0.182 |
| > 20 ng/mL | 400 | 2.049 (1.413 - 2.973) | **< 0.001** |  | 1.390 (0.937 - 2.060) | 0.101 |
| **PI-RADS score** | 1091 |  |  |  |  |  |
| ≤ 3 | 313 | Reference |  |  | Reference |  |
| > 3 | 778 | 1.903 (1.320 - 2.743) | **< 0.001** |  | 1.230 (0.834 - 1.814) | 0.296 |
| **pT** | 1091 |  |  |  |  |  |
| 1-2 | 646 | Reference |  |  | Reference |  |
| 3-4 | 445 | 1.831 (1.366 - 2.456) | **< 0.001** |  | 0.861 (0.594 - 1.249) | 0.431 |
| **pN** | 1091 |  |  |  |  |  |
| 0 | 940 | Reference |  |  | Reference |  |
| 1 | 151 | 2.752 (1.994 - 3.798) | **< 0.001** |  | 1.494 (1.023 - 2.183) | **0.038** |
| **ISUP group** | 1091 |  |  |  |  |  |
| 1 | 161 | Reference |  |  | Reference |  |
| 2 | 299 | 1.490 (0.763 - 2.910) | 0.243 |  | 1.261 (0.640 - 2.484) | 0.503 |
| 3 | 225 | 2.523 (1.324 - 4.810) | **0.005** |  | 1.754 (0.889 - 3.460) | 0.105 |
| 4 | 199 | 2.579 (1.348 - 4.935) | **0.004** |  | 1.579 (0.782 - 3.186) | 0.202 |
| 5 | 207 | 4.450 (2.396 - 8.264) | **< 0.001** |  | 2.185 (1.082 - 4.413) | **0.029** |
| **PSM** | 1091 |  |  |  |  |  |
| Negative | 640 | Reference |  |  | Reference |  |
| Positive | 451 | 1.796 (1.340 - 2.408) | **< 0.001** |  | 1.128 (0.801 - 1.588) | 0.490 |
| **LVI** | 1091 |  |  |  |  |  |
| Negative | 953 | Reference |  |  | Reference |  |
| Positive | 138 | 2.496 (1.772 - 3.515) | **< 0.001** |  | 1.383 (0.939 - 2.039) | 0.101 |
| *P* values in bold are indicative of statistical significance. HR, Hazard Ratio; CI, Confidence Interval, BMI, body mass index; tPSA, total prostate-specific antigen; PI-RADS, Prostate Imaging Reporting and Data System; pT, pathological tumor stage; pN, pathological node stage; ISUP, International Society of Urological Pathology; PSM, positive surgical margin; PNID, perineural invasion density; LVI, lymphovascular invasion. | | | | | | |

| **Supplementary Table 6. Incremental Prognostic Value of PNID via Nested Cox Models** | | | | | | | |
| --- | --- | --- | --- | --- | --- | --- | --- |
| **Endpoint** | **Model** | **Harrell’s C-index (95% CI)** | **ΔC-index  (vs. Model 1)** | ***P*** value | **LRT χ²** | **LRT** P value | **AIC** |
| **BCR** | Model 1 (Base) | 0.707 (0.675–0.742) | Ref | — | Ref | — | 2813.9 |
|  | Model 2 (Model 1 + PNI) | 0.727 (0.695–0.764) | +0.020 | **0.028** | 27.56 | **<0.001** | 2788.3 |
|  | Model 3 (Model 1 + PNID) | 0.765 (0.733–0.800) | +0.058 | **<0.001** | 93.54 | **<0.001** | 2722.4 |
| **rPFS** | Model 1 (Base) | 0.661 (0.626–0.711) | Ref | — | Ref | — | 2234.2 |
|  | Model 2 (Model 1 + PNI) | 0.663 (0.628–0.713) | +0.002 | 0.625 | 1.55 | 0.213 | 2234.6 |
|  | Model 3 (Model 1 + PNID) | 0.667 (0.635–0.717) | +0.006 | 0.309 | 3.44 | 0.063 | 2232.7 |
| BCR, biochemical recurrence; rPFS, radiographic progression-free survival; Ref, reference; LRT, likelihood ratio test; AIC, Akaike Information Criterion. P values for ΔC-index were calculated via paired bootstrap resampling (300 iterations). | | | | | | | |
